# Localization analysis of essential oils in perilla herb (*Perilla frutescens var. crispa*) using derivatized mass spectrometry imaging

**DOI:** 10.1002/fsn3.2232

**Published:** 2021-03-30

**Authors:** Shu Taira, Aya Kiriake‐Yoshinaga, Hitomi Shikano, Ryuzoh Ikeda, Shoko Kobayashi, Kazuaki Yoshinaga

**Affiliations:** ^1^ Faculty of Food and Agricultural Sciences Fukushima University Fukushima Japan; ^2^ Research Center for Food Safety Graduate School of Agricultural and Life Sciences The University of Tokyo Tokyo Japan

**Keywords:** herb, mass spectrometry imaging, matrix‐assisted laser desorption/ionization

## Abstract

The localization of essential oils, including flavor components, in perilla herb (*Perilla frutescens var. crispa*) were visually determined using matrix‐assisted laser desorption/ionization (MALDI) mass spectrometry (MS) imaging. The surface of a perilla leaf was peeled using a cyanoacrylate adhesion compound and contained oil glands that retained their morphology and chemical properties. We imaged the three essential oils perillaldehyde, β‐caryophyllene, and rosmarinic acid (RA). Perillaldehyde was derivatized using glycine to prevent evaporation and allow its detection and imaging while localized in oil glands. β‐caryophyllene also localized in the oil glands and not in the epidermis region. RA was detected throughout the leaf, including the oil glands. Quantitative data for the three essential oils were obtained by gas chromatography‐ or liquid chromatography‐MS. The concentrations of perillaldehyde, β‐caryophyllene, and RA were 12.6 ± 0.62, 0.27 ± 0.02, and 0.16 ± 0.02 [mg/g] in the paste sample of perilla herb. Peeling using a cyanoacrylate adhesion compound, and derivatization of a target such as an aroma component have great potential for mass spectrometry imaging for multiple essential oils.

## Graphical abstract

Perilla herb is well known as food in the whole world. This article visually revealed the localization of fragrance compounds using derivatization imaging MS by avoidance of volatilization.

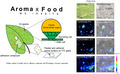

## 1 INTRODUCTION

**FIGURE 1:**
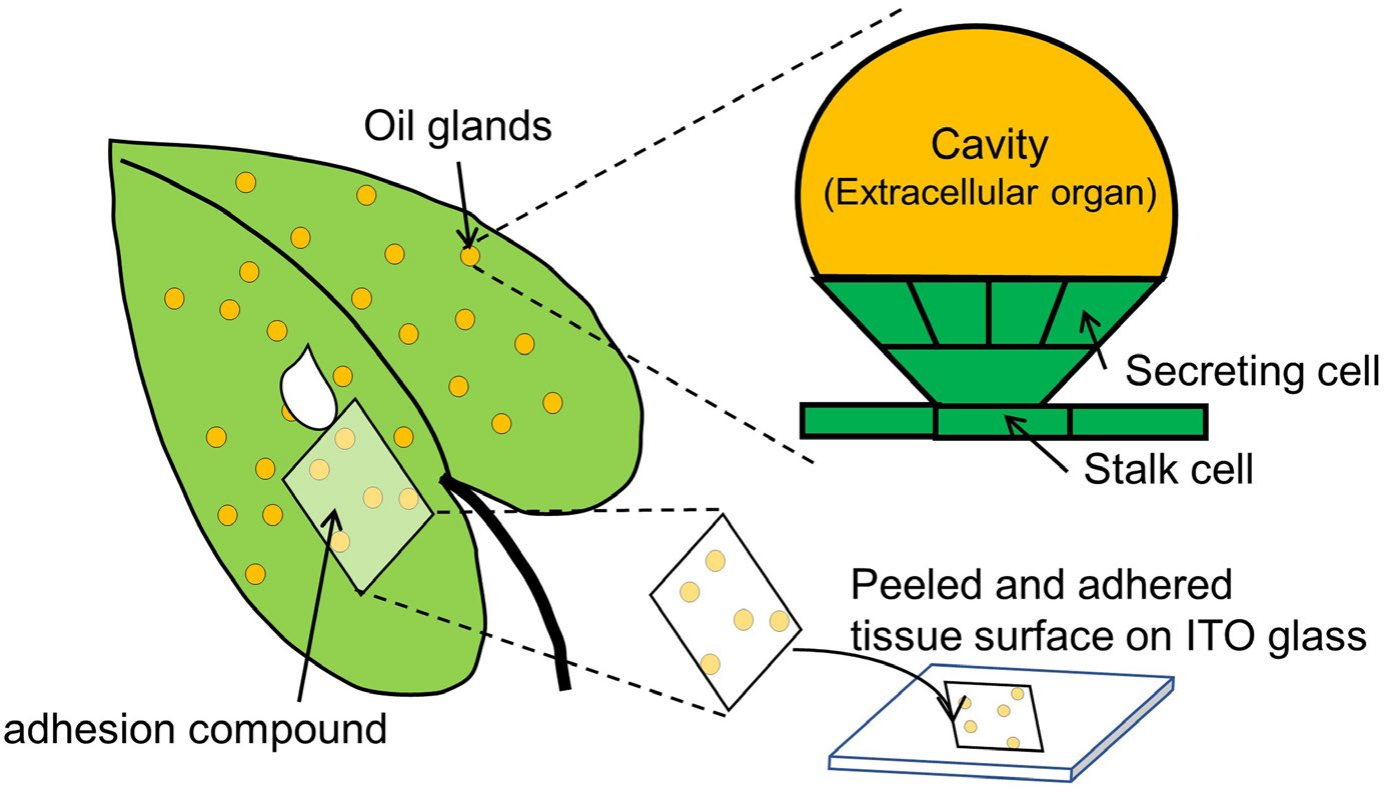
Schematic illustration of perilla herb leaf and the peeling method using an adhesion compound

Perilla herb (*Perilla frutescens var. crispa*) is an annual plant that grows wild and is cultivated for food in many regions in Asia. Aromatic herbs such as perilla contain essential oils such as monoterpenoid perillaldehyde (PA) and bicyclic sesquiterpenes β‐caryophyllene (βC) and have distinctive fragrances. PA and βC also exhibit antibacterial and local anesthetic activity. Perilla herb is thus used as a herbal medicine component (Legault & Pichette, 2007) and may be an antidote for food poisoning. Rosmarinic acid (RA), a common component in perilla herb, has anti‐inflammatory and antioxidant activity, enhances the concentration of monoamine in the brain, and inhibits amyloid‐β aggregation (Hase, 2019). These essential oils act as secondary metabolites in extracellular oil glands in perilla (Figure 1). The oils are produced in inner plant cells and are transported to and stored in the oil glands. Conventional staining methods using Sudan red show the presence of essential oils in oil glands but provides only indirect information as it does not selectively stain essential oils. The construction of antibodies for small molecules is difficult, which limits immunostaining methods for spatial visualization. Gas chromatography (GC)‐MS analysis provides quantitative information and is suitable for volatile organic compounds (VOCs) such as aromatic compounds but provides no visual spatial information. Therefore, information on the localization of these essential oils is lacking and thus the development of selective visualization methods is required not only for fundamental plant science (Shiono & Taira, 2020) but also in fields such as food science (Cho et al., 2020; Enomoto et al., 2018) and omics science (Chen et al., 2019). Mass spectrometry imaging (MSI), and typically matrix‐assisted laser desorption/ionization mass spectrometry (MALDI‐MS), enables the direct mapping and imaging of biomolecules present in tissue sections (Stoeckli et al., 2001). Tissue cross‐sections are mounted on an electrically conductive glass slide, sprayed with an organic matrix, and irradiated with laser shots. Each spot irradiated by the laser becomes a pixel in the final image. Target‐specific markers such as antibodies are not required, and MSI enables the simultaneous detection of multiple analytes in a single section of animal tissue (Fujii, 2018; Hase, 2019; Tatsuta, 2017) or plant tissue (Shikano, 2020; Shiono, 2017; Taira et al., 2015). We solved the difficulty of making sections of thin samples such as leaves by applying cyanoacrylate adhesive to the leaf surface, then peeling the adhesive off the leaf surface with indium tin oxide (ITO) glass (Figure 1). In the present study, focusing on essential oils, we imaged three target compounds (PA (boiling point (BP): 497 [K]), βC (BP: 576 [K]), and RA (BP: 1,194 [K])). Generally, organic compounds with a boiling point below 523[K] are defined as a VOC. Imaging VOCs under vacuum is problematic due to their volatility, and thus, we developed a method of amino acid derivatization by nucleophilic addition reaction of amino group of glycine. The aldehyde group of PA reacts with the primary amine of glycine, increasing its BP value above that of a VOC. We compared the localizations and concentrations of these three essential oils in fresh perilla and perilla paste by MALDI‐MSI and GC‐ and liquid chromatography (LC)‐MS.

## 2 MATERIALS AND METHODS

### 2.1 Sample preparation

Standard (‐)‐perillaldehyde was purchased from Tokyo Chemical Industry. β‐caryophyllene was purchased from Wako. Rosmarinic acid was purchased from Sigma‐Aldrich. Perilla herb (*Perilla frutescens*) was purchased at a supermarket and stored at 4°C prior to extraction and MSI analysis. The sample was also ground in a mortar with a pestle to provide a paste for GC‐ and LC‐MS analysis.

### 2.2 Solid phase micro extraction (SPME) fiber

An SPME fiber (length 10 mm) coated with 50/30 mm divinylbenzene/carbon WR/polydimethylsiloxane (DVB/CAR/PDMS) phase (Restek) was used to extract volatile compounds. The fibers were conditioned before use and thermally cleaned between analyses by inserting the fibers into the injector port of a gas chromatography system set at 270°C for 30 min in a stream of helium.

### 2.3 HS‐SPME/GC‐MS analysis

Headspace SPME that is a clean‐up of procedure was used to extract headspace volatiles from the samples. The *Perilla frutescens* sample was ground in a mortar with a pestle to obtain a paste, and then, 100 mg of paste was placed in a 20 ml headspace vial fitted with a silicone septum. Cyclohexanone (10 µl, 1,000 μg/ml in methanol) as an internal standard was added. After equilibration for at least 10 min, SPME sampling was performed by exposing the fiber for 30 min in the headspace of the sampling vial at 40°C. The SPME device was placed into a GC system equipped with a mass spectrometer (GC: Nexis GC‐2030, MS: TQ8050NX, Shimadzu Corporation) equipped with a capillary column (IntertCap Pure‐WAX, 30 m × 0.25 mm ID, 0.25 μm film, GL Sciences Ltd.). The column temperature was programmed to hold at 40°C for 5 min, subsequently raised to 250°C at 5°C/min, and held for 15 min. The injector temperature was set to 250°C. The flow rate of the carrier gas (helium) was 1.0 ml/min. The MS detector was operated in electron impact ionization mode at 70 eV. The analysis was performed in the SCAN mode in the 30–550 m*/z* range. Tentative identification of constituents was based on comparison of the retention time and mass fragmentation with pure standards, and on computer matching with commercial mass spectra libraries (NIST & WILEY) and a home‐made library based on pure compounds, which were analyzed under identical conditions. Furthermore, the volatile compounds in raw *Perilla frutescens* samples with intact oil glands were analyzed. Briefly, raw *Perilla frutescens* sample (100 mg) was placed in a 20 ml headspace vial fitted with a silicone septum. After the addition of an internal standard, the headspace gas was analyzed by HS‐SPME/GC‐MS under the same analytical conditions as described above. Perillaldehyde content in the *Perilla frutescens* sample was determined by preparing calibration curves of perillaldehyde, with cyclohexanone as an internal standard.

### 2.4 UPLC‐MS analysis

The rosmarinic acid (RA) content in the *Perilla frutescens* sample was determined by UPLC‐MS analysis (ACQUITY UPLC H‐class (Waters, London, UK)) and ESI‐TOF (impact II, Bruker Daltonics)) using an ODS column (BEH C18, 2.1 × 100 mm, 1.7 µm) with the column temperature set at 40°C. Two mobile phases were used for gradient elution: Solvent A, water/formic acid (0.03%); Solvent B, methanol/formic acid (0.03%). Liquid chromatography was carried out at a flow rate of 0.2 ml/min with a linear gradient under the following conditions: 0–2.5 min, solvent A 70%–20%; 2.5–5.0 min, solvent A 0%. RA positive ion was detected using an electrospray ionization mass instrument (impactII, Bruker Daltonics) (end plate offset at 500 V, capillary voltage at 4.5 KV). The RA calibration curve was prepared using an RA standard (5, 25, 50 µg/ml).

### 2.5 Preparation of perilla herb peeling sections for mass spectrometry imaging (MSI)

Cyanoacrylate‐type adhesive compound (Aron Alpha, TOAGOSEI) was dropped on backed perilla herb. A glass slide coated with ITO (Bruker Daltonics) was pressed on the sample to transfer epidermal tissue. The release paper film was pressed on the peeled epidermal tissue to crush the oil glands. Optical images of the tissue were obtained using a microscope scanner (Nanozoomer, Hamamatsu Photonics, Shizuoka, Japan) before analysis by MALDI‐MSI (Supporting information).

### 2.6 MALDI‐MSI

A 10 mg/ml solution of the matrix α‐cyano‐4‐hydroxycinnamic acid (CHCA, Nacalai Tesque) was suspended in 6 ml of acetonitrile/water/trifluoroacetic acid (50/49.9/0.1 *v/v*) and sprayed on the perilla herb tissue sections on the ITO‐coated glass slides (Bruker Daltonics) using an automated pneumatic sprayer (TM‐Sprayer, HTX Tech). Ten passes were sprayed using the following conditions: flow rate, 120 µl/min; air flow, 10 psi; nozzle speed, and 1,100 mm/min.

Ionization and imaging of the essential oils were confirmed with a MALDI‐TOF‐MS (rapifleX, Bruker Daltonics). Tandem MS spectra of the sections were obtained using a collision‐induced dissociation method. Precursor ion (obtained using 1000× shots) and fragment ion (obtained using 4000× shots) signals were integrated using flexControl 3.4 (Bruker Daltonics). Spectra were recorded in positive linear tandem MS mode (ion source 1 voltage, 20 kV; lens voltage, 18 kV). The collision energy was about 1.3 eV, estimated using MALDI‐TOF/TOF (PSD) mode and CID under Ar (manufacturer default settings).

For MSI, the laser spot areas were detected by scanning the sections. The laser spot areas (200 shots) were detected with a spot‐to‐spot center distance of 20 µm in each direction of the *Perilla frutescens* sample. Signals between *m/z* 60–500 were corrected. The peeled tissue surface was irradiated with YAG laser shots in the positive or negative ion detection mode. The laser power was optimized to minimize in‐source decay of the targets. The obtained MS spectra were reconstructed to MS images with a mass bin width of *m/z* ± 0.05 and the pseudo color scale from the mass value using FlexImaging 4.0 software (Bruker Daltonics).

## 3 RESULTS AND DISCUSSION

### 3.1 Detection of essential oils in perilla herb using MALDI‐TOF‐MS and tandem MS

**FIGURE 2:**
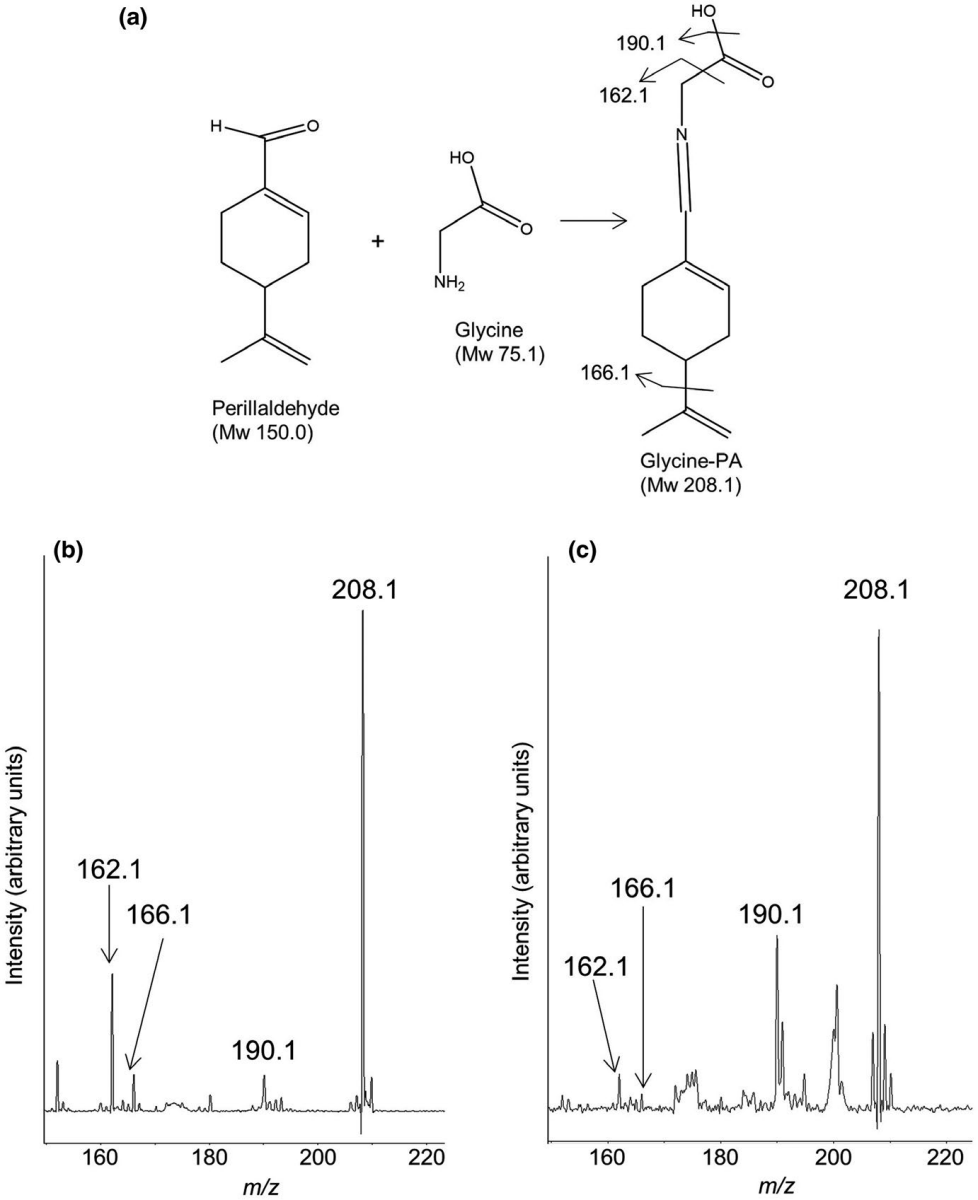
Schematic illustration of the reaction between perillaldehyde (PA) and glycine (a). Tandem mass spectra of glycine‐modified PA (b) standard and (c) perilla section

MALDI‐MS analysis of sample sections detected protonated glycine‐modified PA (Gly‐PA), β‐caryophyllene (βC) and rosmarinic acid (RA) ions at *m/z* 208.1, 205.3 and 360.1, respectively, in positive ion mode. Gly‐PA contains a Schiff base between glycine and PA and showed an increased mass signal (*m/z* 208.1 as protonated signal) (Figure 2a). We confirmed PA modification by glycine by tandem MS using the above‐detected ion as the precursor ion from the standard and from perilla sections (Figure 2b, c). The Gly‐PA precursor ion was cleaved and provided ions corresponding to the glycine hydroxy group and carboxy group, and the lactone ring of PA. As expected from their chemical structures, fragment ions were observed at *m/z* 190.1, *m/z* 166.1 and *m/z* 162.1 from both the standard sample and from perilla sections, suggesting that the peaks of the precursor ion from perilla sections detected by MALDI‐MSI matched the structures of the target analytes.

### 3.2 Quantitative analysis for target molecules in perilla herb

PA and βC standards were separated using the same gradient conditions by GC‐MS (Nexis, GC‐2030, Shimadzu). We selected two different ions that corresponded to PA and βC (*m/z* 67.0 and 93.0). The PA, βC, and RA contents (mg/g sample) were calculated from the peak areas in the chromatograms and by using calibration curves. The concentrations of PA and βC in perilla paste were 12.6 ± 0.62 and 0.27 ± 0.02 [mg/g] sample and in fresh perilla were 0.20 ± 0.07 and 0.03 ± 0.006 [mg/g] sample, respectively. The concentrations of PA and βC in the paste sample were 63 and 9 times that in the fresh sample, showing that essential oils are mostly stored in oil glands. We therefore pressed a peeled perilla herb section to image essential oils. For RA, we used UPLC‐MS since RA is not a VOC. The concentration of RA was 0.16 ± 0.02 [mg/g] sample (Table 1).

**TABLE 1:** Qualification of metabolities of Perilla herb

	Paste (mg/g)		Fresh (mg/g)	
	Average	*SD*	Average	*SD*
Perillaldehyde (497 [K])	12.6^**^	0.62	0.20	0.07
β‐caryophyllene (576 [K])	0.27^**^	0.02	0.03	0.006
Rosmarinic acid (1,194 [K])	0.16	0.02	–	–

Note

(Boiling point) **p < .01 with student's *t*‐test.

### 3.3 MALDI‐MSI of perilla herb sections

**FIGURE 3:**
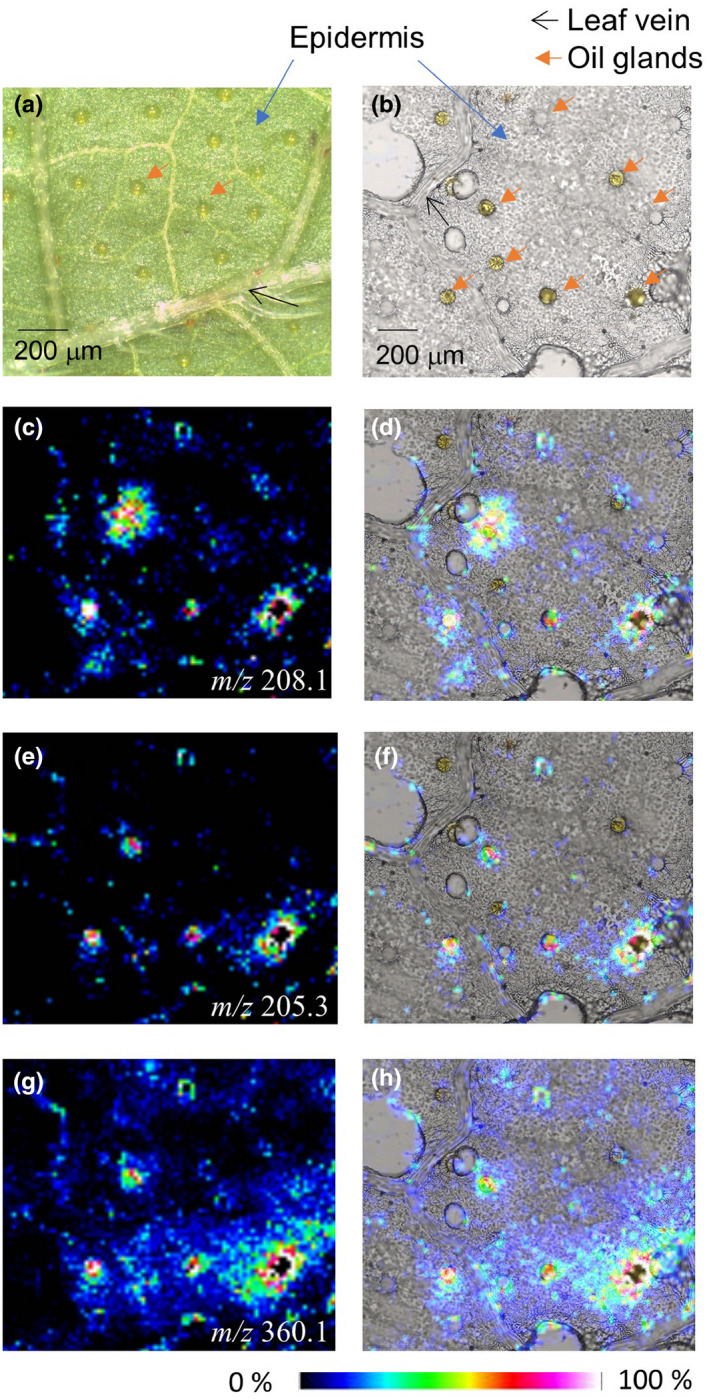
Imaging MS of perilla herb. Optical (a) and microscope (b) images, MS image of gly‐perillaldehyde (*m/z* 208.1) (c) and merged image (d), β‐caryophyllene (*m/z* 205.3) (e) and merged image (f), rosmarinic acid (*m/z* 360.1) (g) and merged image (h)

Figure 3 shows MALDI‐MSI data for three essential oils in perilla herb. The accompanying optical images provide information on perilla herb morphology. The epidermal tissue, epidermis, leaf vein, and oil glands could be observed in perilla herb (Figure 3a) and were confirmed in peeled perilla herb sections (Figure 3b).

Glycine (Gly) was used to derivatize PA after crushing the oil glands in a perilla section using pressure. Thus, PA was imaged by selecting *m/z* 208.1 as the protonated Gly‐PA ion. The image of protonated Gly‐PA was blurred around the crushed oil glands but PA was nonetheless clearly localized at oil glands. A small amount of Gly‐PA was observed in the epidermis region (Figure 3c, d), suggesting that PA is produced in plant cells and stored in oil glands. Organic compounds with a boiling point (BP) below 523[K] are defined as VOCs. Thus, PA (BP: 497 [K]) without glycine modification did not image due to volatilization during measurement whereas Gly‐PA could be imaged. Using chemical information software (ChemDraw Prime 19, Hulinks, Tokyo, Japan), the theoretical boiling point of Gly‐PA was estimated to be 716 [K] and thus Gly‐PA did not volatilize during measurement. The *m/z* 205.3 ion, corresponding to protonated βC, localized at the same region as Gly‐PA, indicating that βC is also contained in oil glands (Figure 3e, f). βC was not derivatized with Gly as it contains no reactive group. The BP of βC (576 [K]) is slightly above that of the VOC threshold (523 [K]). MS measurements were conducted rapidly using the rapifleX MALDI‐TOF‐MS instrument and a 10 [KHz] pulse laser. The measurement area was about 4 [mm^2^] and the laser spot areas (200 shots) were detected with a spot‐to‐spot center distance of 20 µm in each direction on the section. The measurement time was estimated using Equation (1).

(1) $${\left(2,000\;\left[\mu m\right]/20\;\left[\mu m\right]\right)}^{2}\times \left(200/10,000\right)\;\left[s\right]=200\;\left[s\right]$$

The rapid measurement time and borderline VOC BP allowed the imaging of βC in *Perilla frutescens* sections. The estimated BP of RA is below that of VOCs. Protonated RA ion (*m/z* 360.1) was observed throughout the epidermis region, including the oil glands (Figure 3g, h). RA has antibacterial, antifungal, and antioxidant properties and is produced and accumulates in cells as RA‐glycoside (Trócsányi et al., 2020). Plants use RA as an antioxidant. Thus, RA‐glycoside may degrade to RA in the cytoplasm and be transported to oil glands to protect secondary metabolites such as PA and βC. We speculate that oil glands preferentially accumulate metabolites with lower BPs, such as VOCs. PA has a highly reactive aldehyde group. To prevent unnecessary reaction between metabolites, plants may accumulate select molecules in oil glands.

To our knowledge, this is the first study to simultaneously visualize several major metabolites in *Perilla frutescens* tissues. Our imaging results for PA, βC, and RA suggest that these metabolites are stored in oil glands. The aldehyde group in PA reacts with the amino group of Gly to form a Schiff base that prevents volatilization. This approach can aid the investigation of VOCs and demonstrates the utility of derivatization in MS measurements. We determined the distribution of these metabolites in *Perilla frutescens* sections by peeling *Perilla frutescens* surfaces using a cyanoacrylate adhesion compound. The peeled sample surface preserves the morphology and transferred oil glands. Although quantification by MALDI‐MSI is difficult because the signal intensity depends on many factors (e.g., ionization efficiency, extraction efficiency from the tissue, and sample preparation), mass spectrometry imaging is a powerful tool for the direct visualization of fruit compounds and biomolecules in biological tissues (Taira, 2008; Tatsuta, 2017), and may have applications in agriculture relevant to commercial food products.
